# The Impact of Preoperative Sarcopenia on Survival Prognosis in Patients Receiving Neoadjuvant Therapy for Esophageal Cancer: A Systematic Review and Meta-Analysis

**DOI:** 10.3389/fonc.2021.619592

**Published:** 2021-06-23

**Authors:** Sheng-bo Jin, Zi-bin Tian, Xue-li Ding, Ying-jie Guo, Tao Mao, Ya-nan Yu, Kai-xuan Wang, Xue Jing

**Affiliations:** ^1^ Department of Gastroenterology, The Affiliated hospital of Qingdao University, Qingdao, China; ^2^ Department of Gastroenterology, Changhai Hospital, Second Military Medical University/Naval Medical University, Shanghai, China

**Keywords:** sarcopenia, esophageal cancer, neoadjuvant therapy, prognosis, meta-analysis

## Abstract

**Background:**

Sarcopenia is a poor prognostic factor in patients with esophageal cancer (EC). It can be aggravated by neoadjuvant therapy (NAT) that improves the prognosis of patients with EC. Until now, the impact of preoperative sarcopenia on survival prognosis in patients receiving NAT for EC remains unclear.

**Methods:**

We systematically researched relevant studies in the PubMed, EMBASE, Web of Science, the Cochrane Library databases up to March 8, 2020. Prevalence of sarcopenia before and after NAT, overall survival (OS) and disease-free survival (DFS) were collected for analysis. Finally, eleven cohort studies were included.

**Results:**

Pooled analysis indicated that preoperative sarcopenia was negatively associated with OS. (*HR* = 1.290; 95% *CI* [1.078–1.543]; *P* = 0.005; *I* ^2^ = 0.0%) and DFS (*HR* = 1.554; 95% *CI* [1.177–2.052]; *P* = 0.002; *I* ^2^ = 0.0%) in the patients with EC receiving NAT. The prevalence of sarcopenia increased by 15.4% following NAT (95%*CI* [12.9%-17.9%]). Further subgroup analysis indicated that sarcopenia diagnosed following NAT (*HR* = 1.359; 95% *CI* [1.036–1.739]; *P* = 0.015; *I* ^2^ = 6.9%) and age >65 years (*HR* = 1.381; 95% *CI* [1.090– 1.749]; *P* = 0.007; *I* ^2^ = 0.0%) were the independent risk factors for decreased OS.

**Conclusions:**

Clinicians should strengthen the screening of preoperative sarcopenia in patients of EC both receiving NAT and older than 65 years and give active nutritional support to improve the prognosis of patients.

**Systematic Review Registration:**

International Platform of Registered Systematic Review and Meta-Analysis Protocols (INPLASY), identifier INPLASY202050057.

## Introduction

Esophageal cancer (EC) is the sixth leading cause of cancer-related deaths worldwide. The major risk factors include tobacco, alcohol, high body mass index (BMI), and a diet low in fruits (1, 2). Although there have been great advances in surgical and postoperative management techniques that have improved treatment outcomes, the prognosis of EC is still unsatisfactory. It is reported the 3- and 5-year overall survival (OS) rates of the 5283 investigated patients with EC were 49.98% and 39.07% respectively in China (3).

Sarcopenia is a progressive and generalized skeletal muscle disorder involving the accelerated loss of muscle mass and function. Recently, numerous studies have shown that the poor prognosis of many malignant tumors is associated with sarcopenia (4–6). The patients with EC are more likely to suffer from sarcopenia due to malnutrition caused by esophageal stenosis and poor oral intake. Radical esophageal resection is one of the most effective treatments for EC, but long-term outcomes of the patients with surgery alone are unsatisfactory. The addition of adjuvant chemotherapy can improve OS and the disease-free survival (DFS) (7, 8). However, skeletal muscle mass and strength may furtherly be reduced during NAT (9), because of chemotherapy-related toxicities (10), disease progression (11) and adverse postoperative complications (12). Until now, whether sarcopenia would influence the survival rate of EC patients remains controversial (13, 14). Therefore, we aim to assess the effects of sarcopenia on the prognosis of EC patients undergoing NAT in this meta-analysis.

## Methods and Materials

We investigated relevant studies from PubMed, Embase, the Cochrane library, and Web of Science up to March 8, 2020. Key words used in our searches include the following: (esophageal neoplasms OR esophagus neoplasm OR cancer of esophagus OR esophagus cancer OR esophageal cancer) AND (sarcopenia OR sarcopenic OR skeletal muscle depletion OR muscle index OR muscle mass) AND (esophagectomy OR surgery or surgical OR resection) AND (neoadjuvant therapy OR neoadjuvant treatment OR neoadjuvant chemoradiotherapy OR neoadjuvant chemotherapy OR neoadjuvant radiotherapy OR preoperative chemotherapy OR preoperative radiotherapy preoperative chemoradiotherapy OR preoperative therapy). We manually verified for additional studies based on references used the retrieved articles. The registration number is INPLASY202050057. The DOI number is 10.37766/inplasy2020.5.0057.

### Study Selection

The valid diagnostic criteria for sarcopenia remain controversial (15). The European Working Group on Sarcopenia suggested (16) CT image analysis is considered as gold standards for non-invasive assessment of muscle quantity/mass. Hence, this meta-analysis focuses on the studies using SMI defined sarcopenia.

In this study, we established following inclusion criteria: 1) studies of EC patients who underwent radical esophagectomy, 2) studies of EC patients received preoperative NAT (including chemotherapy and chemoradiotherapy), 3) studies with sufficient data of OS and DFS, 4) studies of patients describing definite time to diagnosis of sarcopenia (before NAT or after NAT), 5) SMI is used as the diagnostic standard of sarcopenia. The exclusion criteria were as follows: 1) case reports, reviews, conference abstracts or preclinical studies, 2) studies citing literature with incomplete data, and 3) nonhuman studies.

### Data Extraction

We assigned two authors independently to search for relevant studies and screen the literature using titles and abstracts. After the initial screening, the full text of the articles that satisfied the inclusion criteria were evaluated. In this process, two authors collected data from the included literature, compared the outcome data, and resolved conflicts through discussion and consensus. The following information was extracted from these studies: last name, publication year, country of the patients, research type, number of patients, patient age, follow-up time, diagnosis time of sarcopenia, prevalence of sarcopenia before neoadjuvant therapy and after neoadjuvant therapy, hazard ratio (*HR*) of patients for OS and DFS with 95% confidence interval (*CI*), and *P*-value. When data could not be extracted, we used the Engauge Digitizer 10.8 software to extract survival data from the Kaplan-Meier curves. Following data extraction, meta-analysis was conducted using STATA software version 15.0 (Stata Corp, College Station, TX, USA) to combine the OS and DFS (17), and the outcomes were calculated according to the method described by Parmar (18). All statistical tests were bilateral, and a *P* value < 0.05 was regarded as statistically significant. The heterogeneity of the pooled results was assessed through Cochran’s Q test and Higgins I-squared statistic. Random effects models were applied when significant heterogeneity was identified by *I^2^* >50%, otherwise fixed effects models were utilized. Subgroup analysis was performed based on the time of diagnosis of sarcopenia and patient’s age. Begg’s funnel plot and sensitivity analysis were used to assess publication bias. Sensitivity analyses were performed to evaluate the overall results after omission of specific studies. A two-sided *P* value <0.05 was defined as statistically significant.

### Quality Assessment

We evaluated the quality of the data *via* the Newcastle-Ottawa Scale (NOS) (19). Three factors were used for this evaluation: 1) patient selection, 2) comparability of research groups, and 3) assessment of outcomes. This quality assessment scale had a maximum score of 9, and studies with scores ≥ 7 were considered high quality.

## Results

**Figure 1:**
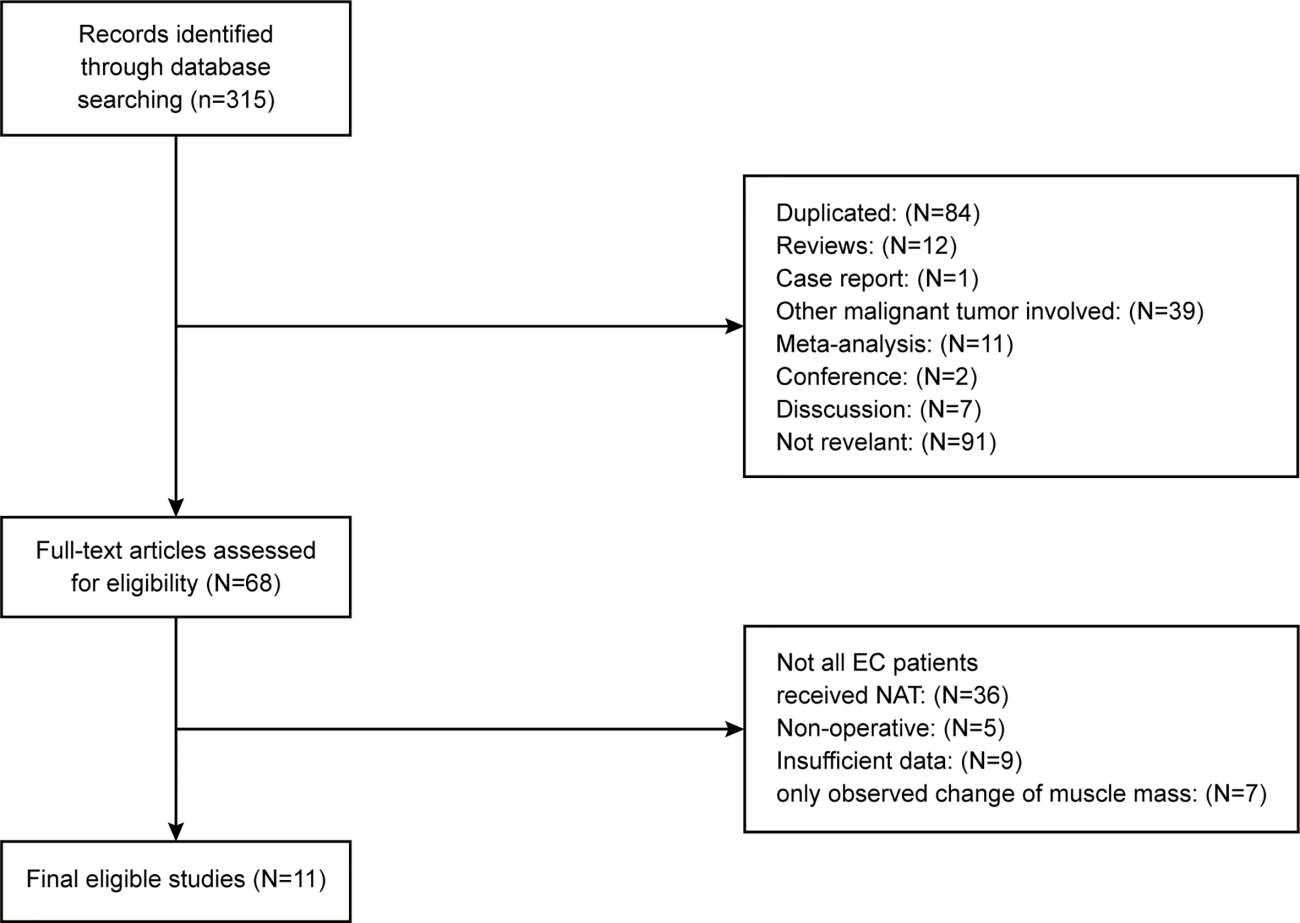
Methodological flow diagram of the meta-analysis.

The basic characteristics of the included studies are shown in **Tables 1** and **2**. Ultimately, we selected eleven cohort studies from the electronic database. The results of these studies were shown in the flow diagram (**Figure 1**). A total of 315 studies were included based on the search strategy, and 68 studies were selected for detailed evaluation following title and abstract screening. Partial studies were furtherly excluded for the following reasons: not all patients received NAT (28), non-operative (5), and only observed change of muscle mass but not diagnosed sarcopenia (4). Moreover, some studies (9) were further excluded due to a lack of relevant data extracted for analysis (including the number of patients, missing survival curves/data, and inconsistent diagnostic criteria) (3). Finally, a total of eleven cohort studies (1485 patients) were included in the meta-analysis. The timing of sarcopenia diagnosis varied among the included studies: before NAT (5), after NAT (5), or two time periods measured (1). Six studies reported prevalence of sarcopenia before or/and after neoadjuvant therapy, all eleven studies reported OS, but only three studies reported DFS (11, 20, 25). The follow-up time was relatively short with a median time ranging from 11 to 39 months, and some studies did not provide a follow-up time (14, 23, 25).

**Table 1:** Characteristics of the included studies in this meta-analysis.

Author	Year	Country	Sample size	Sex (female%)	Age	Follow- up (month)	SarcopeniaDefinition	Sarcopenia	Non-sarcopenia	Pre-NACT(%)	Post-NACT (%)	NACT
Elliott et al. (11) 60	2017	Ireland	192	20.3%	61.6 ± 9.3	Median: 26	man: <52.4 cm^2^/m^2^ woman: <38.5 cm^2^/m^2^	49	143	15.8%	30.8%	nCT or nCRT
Paireder et al. (20) 100	2017	Austria	130	18.5%	61.4 (30.8–81.0)	Median: 21.5	man:<55cm^2^/m^2^ woman:<39m^2^/m^2^ for	80	50	42	57.7%	nCT or nCRT
Panje et al. (13) 72	2019	Switzerland	300	12.3%	61 (36–75)	Median: 48	man:<43m^2^/m^2^(BMI < 25 kg/m^2^), 53 cm^2^/m^2^ (BMI ≥ 25 kg/m^2^); women: 41 cm^2^/m^2^	239	61	29.5%	63.9%	nCRT
Saeki et al. (14) 60	2018	Japan	157	16.7%	64.9	NA	man:<47.27 cm^2^/m^2^ woman: <36.91 cm^2^/m^2^	85	72	41.4%	59.2%	nCRT
Yip et al. (21) 60	2014	UK	35	14%	63 (34–78)	Median: 24	man: <52.4 cm^2^/m^2^ woman: <38.5 cm^2^/m^2^	15	20	26%	43%	nCRT
Ma et al.-1 (22) 125	2019	Switzerland	174	8%	67 (36–91)	Median: 11–38	man: <55 cm^2^/m^2^ woman: <39cm^2^/m^2^	101	73	58.0%	72.4%	nCRT
Ma et al.-2 (22) 125	2019	Switzerland	174	8%	67 (36–91)	Median: 11–38	man: <55 cm^2^/m^2^ woman: <39 cm^2^/m^2^	126	48	58.0%	72.4%	nCRT
Tan et al. (23) 83	2014	UK	89	24.7%	65.8 ± 8.1	NA	man: < 52.4 cm^2^/m^2^ woman: <38.5 cm^2^/m^2^	44	45	44.9%	NA	nCT
Grotenhuis et al. (24) 60	2016	Netherlands	120	27%	62 (19–78)	Median: 20	man: <52.4 cm^2^/m^2^ woman: <38.5 cm^2^/m^2^	54	66	NA	54%	nCRT
Huang et al. (25) 40	2020	China	107	5.6%	54.1 ± 7.5	NA	Man:<52.4 cm^2^/m^2^ woman: <38.5 cm^2^/m^2^	65	42	60.7%	NA	nCRT
Jarvinen et al. (26) 24	2018	Finland	115	25.2%	63 ± 9	at least 24 months	man: <52.4 cm^2^/m^2^ woman: < 38.5 cm^2^/m^2^	92	23	NA	80%	nCRT
Mayanagi et al. (27) 36	2017	Japan	66	13.6%	63.3 ± 8.0	Median: 39	man: <52.4 cm^2^/m^2^ woman: <38.5 cm^2^/m^2^	NA	NA	NA	NA	nCT

**Table 2:** Main outcomes extracted from the studies included in our meta-analysis.

First Author	Sarcopenia diagnosis time	Study design	QualityAssessment (NOS)	OS		*P* Value	DFS		*P* Value
				HR	95%CI		HR	95%CI	
Elliott	Post-NACT	Cohort study	7 stars	1.44	0.92–2.24	0.11	1.40	0.90–2.18	0.14
Paireder	Post-NACT	Cohort study	7 stars	1.31	0.79–2.18	0.036	1.65	0.97–2.81	0.65
Panje	Post-NACT	Cohort study	8 stars	0.68	0.21–2.26	0.72	NA	NA	NA
Saeki	Pre-NACT	Cohort study	6 stars	0.88	0.50–1.57	0.6875	NA	NA	NA
Yip	Post-NACT	Cohort study	8 stars	1.74	0.50–6.02	0.063	NA	NA	NA
Ma-1	Pre-NACT	Cohort study	7 stars	1.27	0.84–1.94	0.254	NA	NA	NA
Ma-2	Post-NACT	Cohort study	7 stars	1.92	1.18–3.13	0.007	NA	NA	NA
Tan	Pre-NACT	Cohort study	6 stars	1.42	0.71–2.84	0.04	NA	NA	NA
Grotenhuis	Pre-NACT	Cohort study	7 stars	0.90	0.46–1.77	0.77	NA	NA	NA
Huang	Pre-NACT	Cohort study	7 stars	1.71	0.78–3.71	<0.001	1.67	1.04–2.71	0.02
Jarvinen	Post-NACT	Cohort study	6 stars	0.65	0.28–1.50	0.74	NA	NA	NA
Mayaagi	Pre-NACT	Cohort study	7 stars	2.56	0.60–10.8	0.202	NA	NA	NA

nCRT, Neoadjuvant chemoradiotherapy; nCT, Neoadjuvant chemotherapy; OS, Overall survival; DFS, Diseasefreesurvival; HR, Hazard ratio.

### Impact of Preoperative Sarcopenia on the Survival of Patients Receiving Neoadjuvant Therapy for Esophageal Cancer

**Figure 2:**
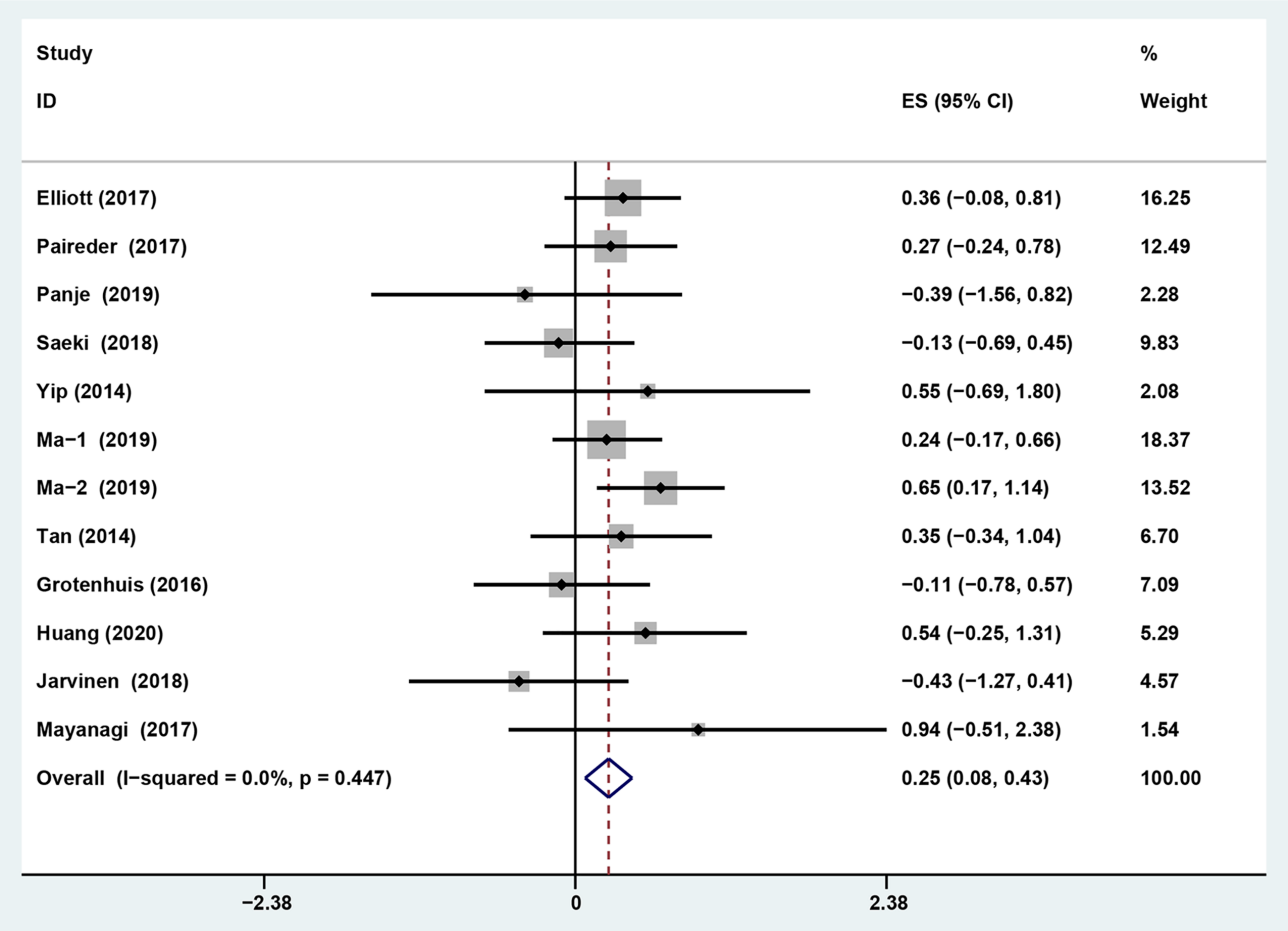
Forest plot of preoperative diagnosis of sarcopenia in esophageal cancer patients undergoing neoadjuvant therapy for overall survival.

**Figure 3:**
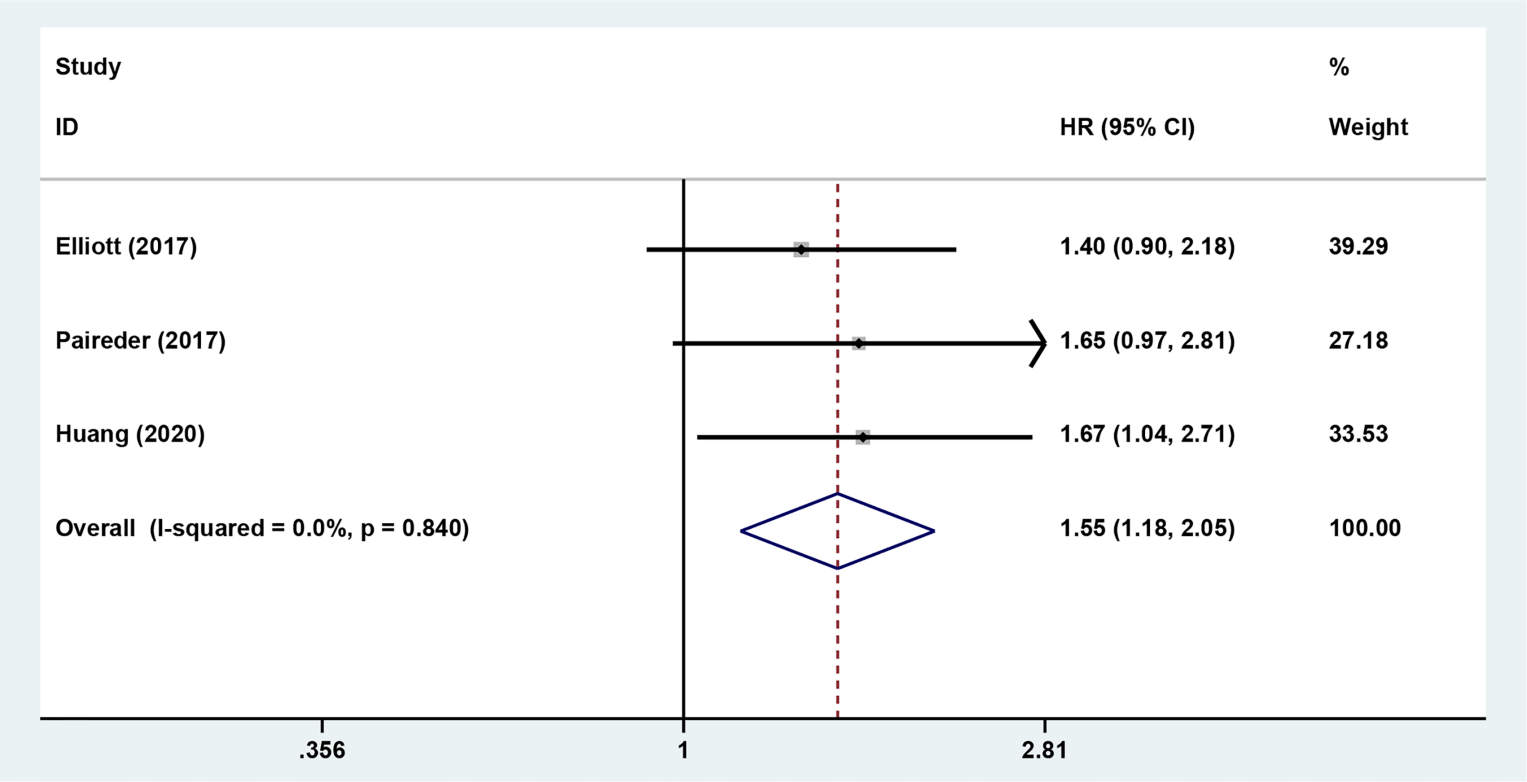
Forest plot of preoperative diagnosis of sarcopenia in esophageal cancer patients undergoing neoadjuvant therapy for disease free survival.

The results showed that preoperative sarcopenia was an independent unfavorable predictor for the prognosis of EC patients received NAT (fixed effects models: *HR*= 1.290; 95% *CI* [1.078–1.543]; *Z* = 2.78; *P* = 0.005; *I* ^2^ = 0.0%) (**Figure 2**). DFS was also significantly related to sarcopenia (fixed effects models: *HR* = 1.553; 95% *CI* [1.177–2.049]; *P* = 0.002; *I* ^2^ = 0.0%) (**Figure 3**).

**Figure 4:**
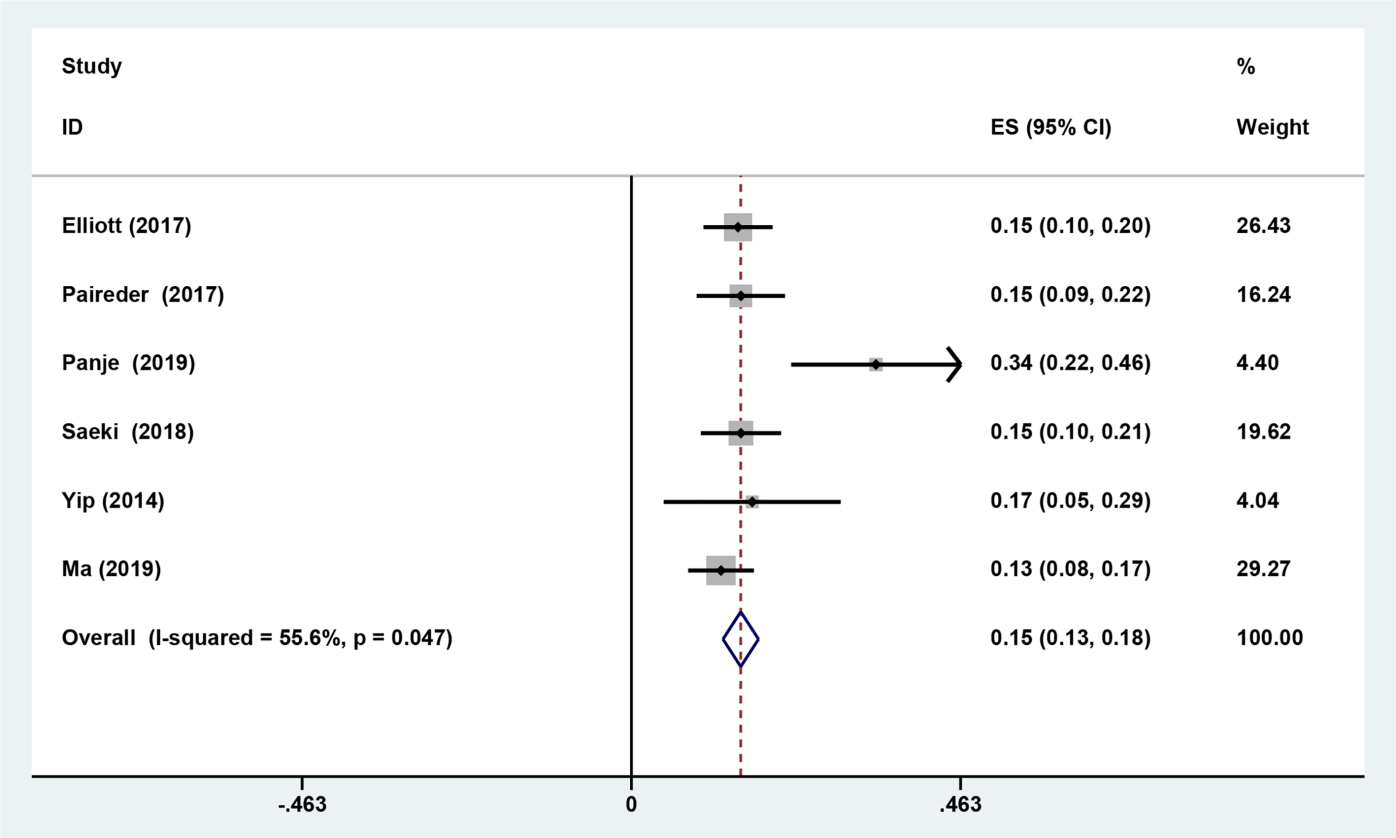
The difference in the prevalence of sarcopenia before and after neoadjuvant therapy.

**Figure 5:**
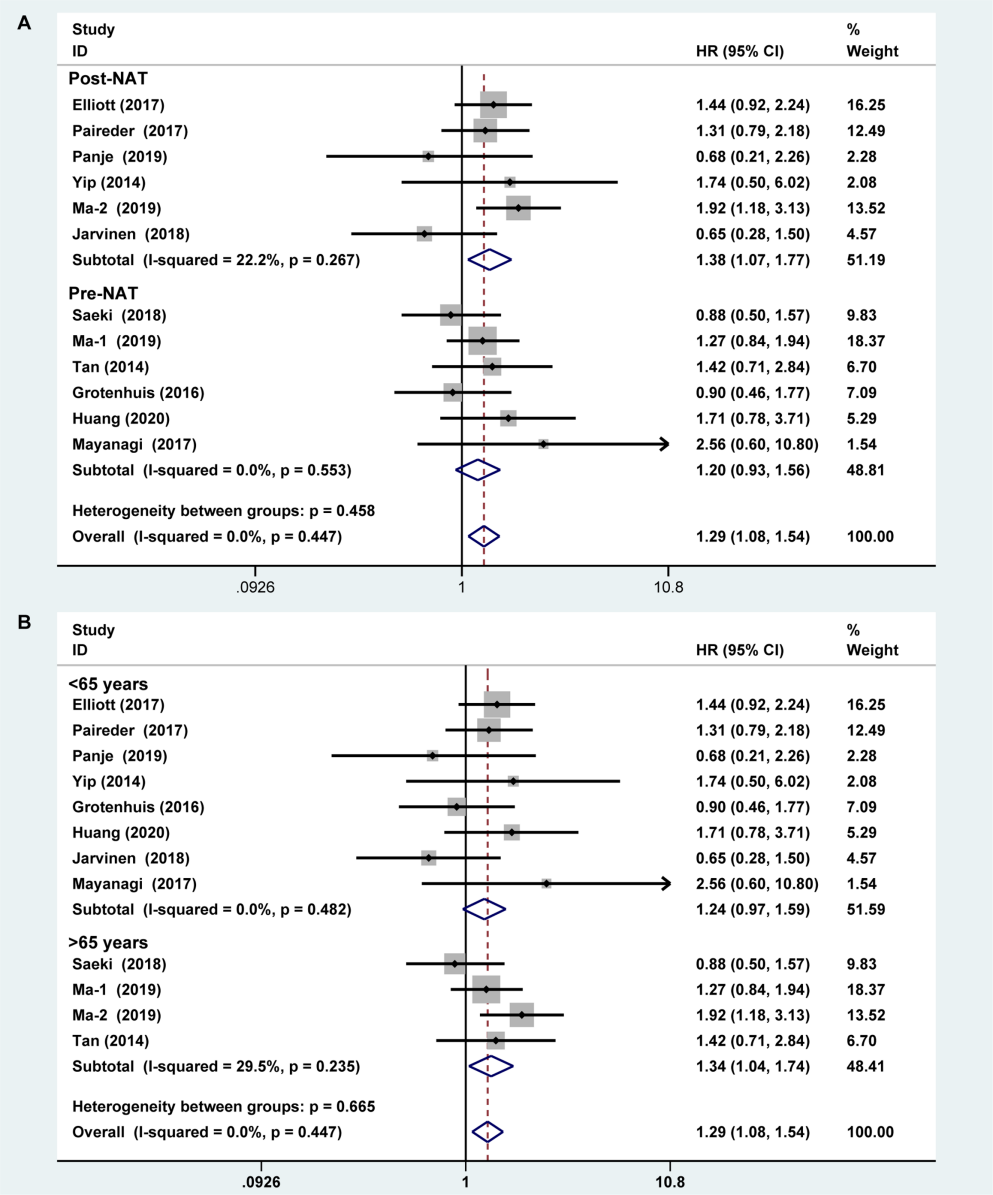
Subgroup analysis of overall survival based on sarcopenia diagnosis time **(A)** and age of patients **(B)**. Note: sarcopenia diagnosis time included before neoadjuvant therapy and after neoadjuvant therapy. For age of patients, the cut-off value was 65 years.

Six studies reported the difference in the prevalence of sarcopenia before or/and after NAT and the results shown that the incidence of patients with sarcopenia increased to 15.4% (95%*CI* [12.9%-17.9%]) after receiving NAT (**Figure 4**). This finding indicated that skeletal muscle mass of the EC patients decreased significantly after neoadjuvant therapy. Therefore, we further divided patients into the before NAT group and the after NAT group. There was no statistically significant difference about OS in the before NAT group (fixed effects models: *HR* =1.290; 95% *CI* [1.078–1.543]; *Z* = 1.41; *P* =0.158; *I* ^2^ = 0.0%) (**Figure 5A**), but the other group was opposite. The patients diagnosed with sarcopenia following NAT had a poor prognosis (fixed effects models: *HR* = 1.378; 95% *CI* [1.073–1.771]; *Z* =2.51; *P* = 0.012; *I* ^2^ = 22.2%).

Sarcopenia also have a significant correlation with age, which also an important risk factor for OS (29, 30). We analyzed the relationship between age and OS for EC patients who diagnosed preoperative sarcopenia and received NAT. The patients were divided into two subgroups by the age (<65 years and >65 years; age based on average or median). The results showed that patients were worse OS with sarcopenia in the group of older than 65 years, which is a significant statistical difference (fixed effects models: *HR* = 1.344; 95% *CI* [1.038–1.739; *Z* = 2.25; *I^2^* = 29.5%; *P* = 0.025) (**Figure 5B**).

### Publication Bias

**Figure 6:**
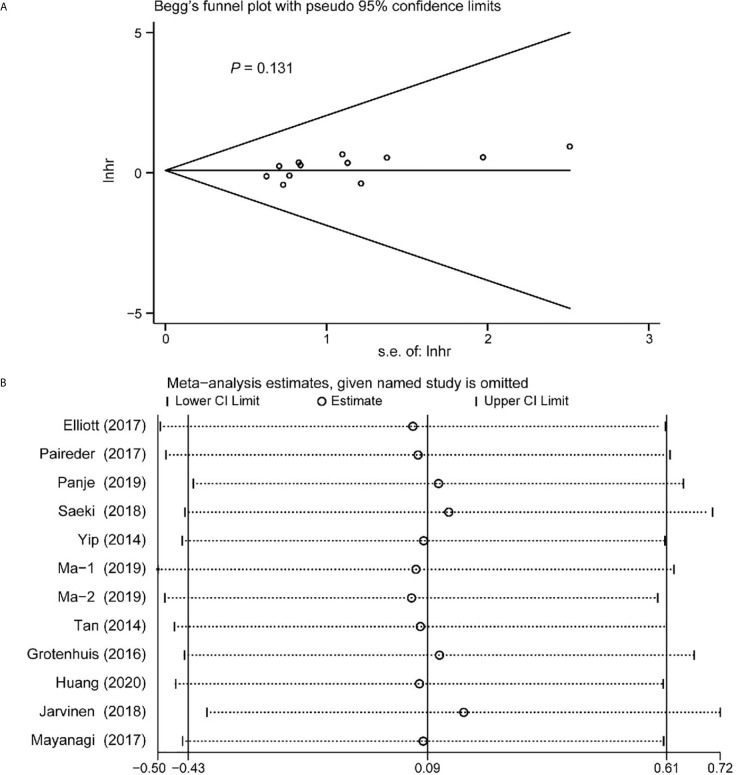
Funnel plot of the all the included studies for the analysis of overall survival. Begg’s test (*P* = 0.131) **(A)** and sensitivity analysis **(B)**.

**Figure 7:**
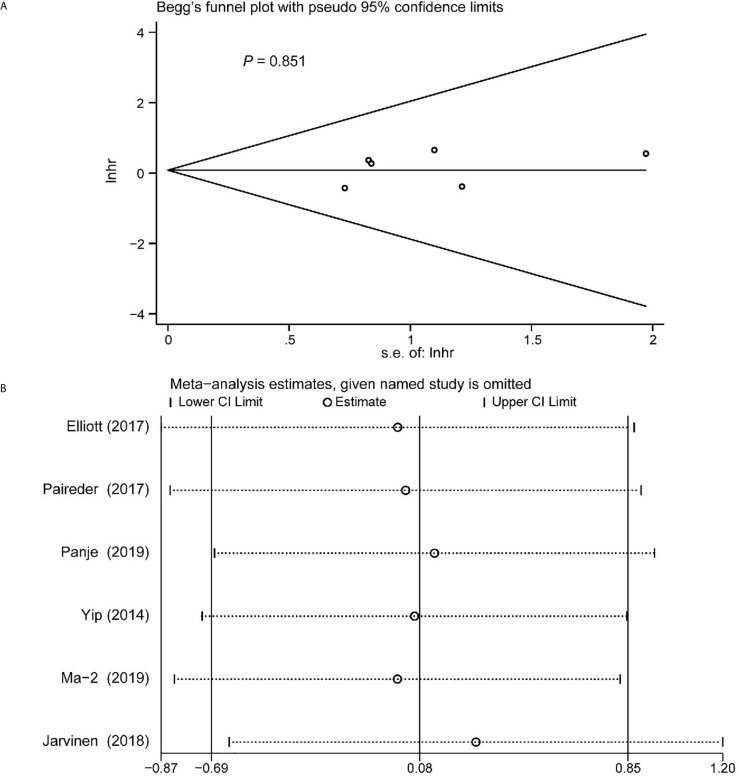
Funnel plot of the included following NACT studies for the analysis of overall survival. Begg’s test (*P* = 0.851) **(A)** and sensitivity analysis **(B)**.

**Figure 8:**
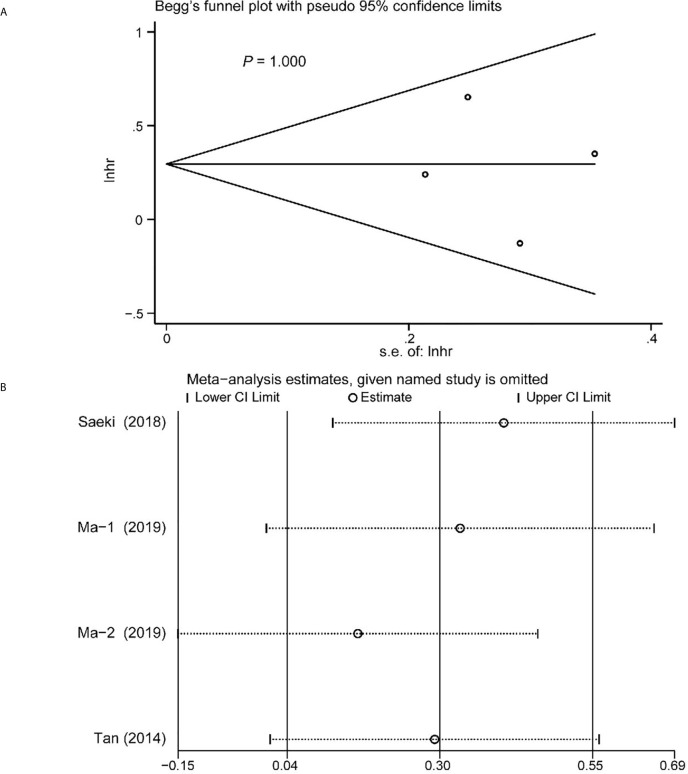
Funnel plot of the included>65 years of age studies for the analysis of overall survival. Begg’s test (*P* = 1.000) **(A)** and sensitivity analysis **(B)**.

Begg’s funnel plot and sensitivity analysis performed to estimate the potential publication bias for all the studies of OS and the studies of OS of patients diagnosed with sarcopenia following NACT. The *P* values were 0.131(Begg’s test) (**Figure 6A**), 0.851 (Begg’s test) (**Figure 7A**) and 1.000 (Begg’s test) (**Figure 8A**), which suggested no publication bias (**Figures 6B**–**8B**).

## Discussion

Sarcopenia is characterized by a decline of skeletal muscle quantity/mass, even the muscle function. However, a uniform criteria of sarcopenia is controversial. Some studies consider that sarcopenia defined by using CT image analysis is a valid, accurate, and precise method. The skeletal muscle index (SMI) is often used as diagnostic index. The European Working Group on Sarcopenia also suggests (16) CT image analysis is considered as gold standards for non-invasive assessment of muscle quantity/mass. Hence, we take the SMI as the diagnostic criterion reducing the inconsistency of diagnostic criteria in this meta-analysis.

Nowadays, NAT has been applied in a mass of malignant tumors because of great advantages such as inducing tumor regression, early treatment of micro metastatic lesions, reducing the risk of R1 resection (31). However, NAT may affect patients’ nutritional status because of treatment-related toxicities and other contributing factors. Whether NAT may worsen survival prognosis by increasing sarcopenia is unclear. Therefore, we investigated the impact of sarcopenia on the prognosis of EC patients undergoing NAT prior to radical surgery.

This meta-analysis found that sarcopenia following NAT had an adverse impact on long-term survival outcomes of EC patients, which was consistent with other reports (9, 32, 33). However, there was a novel additional suggestion in the study. By the subgroups analysis according to the diagnosis time of sarcopenia and the age, the results showed that the patients of EC with sarcopenia after receiving NAT followed by surgery had a worse prognosis, as well as the patients of older than 65 years. Wang et al. (34) reported that NAT was an independent risk factor for sarcopenia, but the impact of NAT for survival prognosis of EC patients was not addressed. In addition, Deng et al. (35) showed that NAT affected the survival prognosis of EC patients, but it did not consider the change of skeletal muscle mass during NAT. The prevalence of sarcopenia inevitably rises with age, while the average mass or strength muscle declines (36). This study showed that patients older than 65 years with sarcopenia were associated with worse OS, which was meaningful and different from other similar reports. Some study showed sarcopenic obesity was an independent predictor of prognosis in elderly patients (>70 years) received NAT followed by surgery for elderly cStage II/III esophageal squamous cell carcinoma (ESCC) (37). But these patients often had little change in weight and were not easy to be diagnosed with sarcopenic obese. EC patients with sarcopenic obese were a higher risk for developing DLT (dose limiting toxicity) during chemotherapy compared with the patients with sarcopenia only. EC patients with sarcopenic obese maybe have a poorer prognosis.

Recent studies reported that skeletal muscle loss is closely correlated with febrile neutropenia and grade neutropenia (38). One of the reasons is that systemic inflammation due to febrile neutropenia (12). The reduction in skeletal muscle mass is caused by an imbalance in protein metabolism, which is characterized by a significantly smaller muscle fiber cross-sectional area. Sarcopenia is also associated with a higher risk of toxicity in EC patients undergoing NACT (32); however, the underlying mechanisms are still unclear. The activation of UPS, IGF-1, and the NF-κB signaling pathway plays a major role in inducing skeletal muscle atrophy (39). Furtherly, cancer anorexia (40) and severe dysphagia (41) aggravate the sarcopenia.

This study provides advice that clinical physicians should pay more attention to assess nutritional status following NAT in the EC patients. Allum et al. (28) conclude that EC patients with nutritional risk should be given 10-14 days of nutritional support before operation. Several approaches have been used to nutritional support during neoadjuvant therapy including esophageal stenting, jejunostomy or gastrostomy, and nasogastric or nasojejunal feeding (42–44). These approaches may improve prognosis.

There are some limitations in this meta-analysis. First, we have not found relevant randomized controlled clinical trials (RCTs). Therefore, we summarized and analyzed the current cohort studies, which may be potentially biased regarding the prognosis estimate. Then, we assessed the quality of the included studies in order to decrease the bias by NOS. Second, we could not directly obtain HRs for OS and DFS from some studies. The result of OS or DFS were calculated by the Engauge Digitizer. Third, the different diagnostic range of SMI among these studies may lead a bias of results, because of no unified agreed upon criteria for the sarcopenia by CT analysis. Finally, with the advancement of medicine, more and more drugs, technologies, and immunotherapy are used to treat esophageal cancer, and neoadjuvant treatment options are also diverse. Such diversity makes it exceedingly difficult to implement a unified neoadjuvant therapy.

In conclusion, we conducted this comprehensive meta-analysis to assess the impact of preoperative sarcopenia on survival prognosis in patients receiving NAT for esophageal cancer. We noted that EC patients received NAT and diagnosed preoperative sarcopenia had an obviously worse OS and DFS than those patients who were not diagnosed preoperative sarcopenia. The older age (>65 years) and sarcopenia following NAT were independent risk factors for OS. Thus, clinicians should strengthen the screening of preoperative sarcopenia in patients of EC both receiving NAT and older than 65 years and give active nutritional support to improve the prognosis of patients. However, more large scale, well-designed, high-quality prospective RCT studies are required to confirm these conclusions in the future.

## Data Availability Statement

The original contributions presented in the study are included in the article/supplementary material. Further inquiries can be directed to the corresponding authors.

## Author Contributions

S-bJ and XJ contributed to study conception and design. S-bJ and Y-jG collected the data. S-bJ, Y-jG, K-xW, and XJ analyzed and interpreted the data. S-bJ wrote the manuscript. Z-bT, XJ, TM, X-lD, Y-nY, and K-xW made critical revisions to the article. All authors contributed to the article and approved the submitted version.

## Funding

This work was supported by China Postdoctoral Science Foundation (Grant No. 2019M652332).

## Conflict of Interest

The authors declare that the research was conducted in the absence of any commercial or financial relationships that could be construed as a potential conflict of interest.
